# How are Unmanned Aerial Vehicles (UAVs) Revolutionizing Forest Operations? A Systematic Review of Current Applications and Future Opportunities

**DOI:** 10.1007/s00267-026-02536-8

**Published:** 2026-06-27

**Authors:** Prakash Ojha, Marissa ‘Jo’ Daniel

**Affiliations:** College of Forestry, Wildlife and Environment, Auburn University, Auburn, AL USA

**Keywords:** UAVs, Forest operations, Drone, Harvesting, Logging, Remote sensing, Machine learning

## Abstract

The use of unmanned aerial vehicles (UAVs), or drones, in forest operations has increased significantly, with applications ranging from rut and deformation mapping and canopy and gap analysis to road geometry and stockpile/woodchip volume calculations. However, no comprehensive reviews have synthesized the use of UAVs in forest operations. Here we present a systematic review of 48 studies on UAV use in harvesting, logging, site preparation, and forest roads. PRISMA guidelines were used to collect and analyze the literature from the Web of Science (WoS) and Scopus databases. The review reported five major forest operation domains, among which soil disturbance and erosion management received the greatest number of studies, followed by forest harvesting and impact monitoring, whereas UAVs are least commonly applied to forest roads and infrastructure, which remains comparatively understudied. Most used UAVs are multirotor, such as the DJI Phantom 4 Pro equipped with RGB sensors, with Agisoft Metashape being the most common image processing software. Overall, UAV-based methods have been reported to be more efficient, accurate, and cost-effective than traditional surveys. Key limitations include limited battery life, susceptibility to obstacles, and longer processing times. Since 2020, the use of UAVs has increased significantly, peaking in 2023, with the United States and Croatia contributing the most publications. The integration of UAV images with advanced technologies, such as artificial intelligence, is emerging and offers new opportunities for operational monitoring and decision support. Our findings demonstrate that UAVs are essential tools for forest operations, although further technological advancements and improvements are necessary to enhance their effectiveness and overcome current limitations.

## Introduction

Unmanned aerial vehicles (UAVs), commonly known as drones, are remotely piloted platforms that are increasingly used as remote sensing tools in various forestry applications (Ecke et al. 2022). UAVs have accelerated forest assessment and management by providing rapid, cost-effective (Ota et al. 2019) and high-resolution spatial data (Iheaturu et al. 2024) that are important for mapping, monitoring, and managing forest resources (Zhao et al. 2023; Buchelt et al. 2024).

Forest operations include activities such as felling, skidding, hauling, and site preparation. The use of UAVs in forest operations has significantly increased in multiple domains, ranging from soil disturbance and erosion, harvest impact monitoring, and residual biomass, to forest road engineering. The UAV-derived point clouds, orthomosaics, and surface models are used to map the depth of wheel ruts and surface deformation caused by machines, estimate the soil erosion rates (Parajuli et al. 2025), delineate canopy gaps (Ota et al. 2019), evaluate regeneration, detect harvesting traces, and measure the forest road cut volume (Papa et al. 2025). The integration of UAV imagery with advanced technologies, such as artificial intelligence (AI), particularly machine learning (ML) and deep learning (DL), has been observed. Similarly, the use of advanced sensors, such as LiDAR, has also increased over the past few years.

Several reviews have examined UAVs in forestry, including but not limited to forest health monitoring (Ecke et al. 2022), general applications (Iglhaut et al. 2019), and data collection practices (Guimarães et al. 2020; Pádua et al. 2017). However, there is no comprehensive, operations-focused review that synthesizes UAV applications in forest operations and engineering. Key questions, therefore, remain unanswered:

1. Which type of operation (e.g., soil disturbance, harvest impact monitoring, residual biomass, productivity modeling, and forest road engineering) has been most widely studied?
2. What types of UAV models, sensors, and image/flight processing software are being utilized in operational contexts?
3. What are the major challenges and limitations, and what are the opportunities for future research?
4. How have publication trends evolved across regions?

To address these gaps, this study conducts a systematic review of peer-reviewed literature that reports the use of UAVs in forest operations and engineering activities, providing a comprehensive overview of the application of UAVs in forestry operations research. We synthesize existing research, identify key UAV models and sensors, and evaluate geographic and temporal trends in application and methodological advances. We also highlight major challenges and limitations, and outline future research directions and opportunities. This review aims to provide a practical understanding of integrating UAVs into forest operations, including recommendations on technology adoption and workforce development to forest industry officials, loggers, researchers, and practitioners. By doing so, it can support more efficient and sustainable forest resource utilization and management.

## Materials and Methods

### Literature Search Strategy

**Fig. 1:**
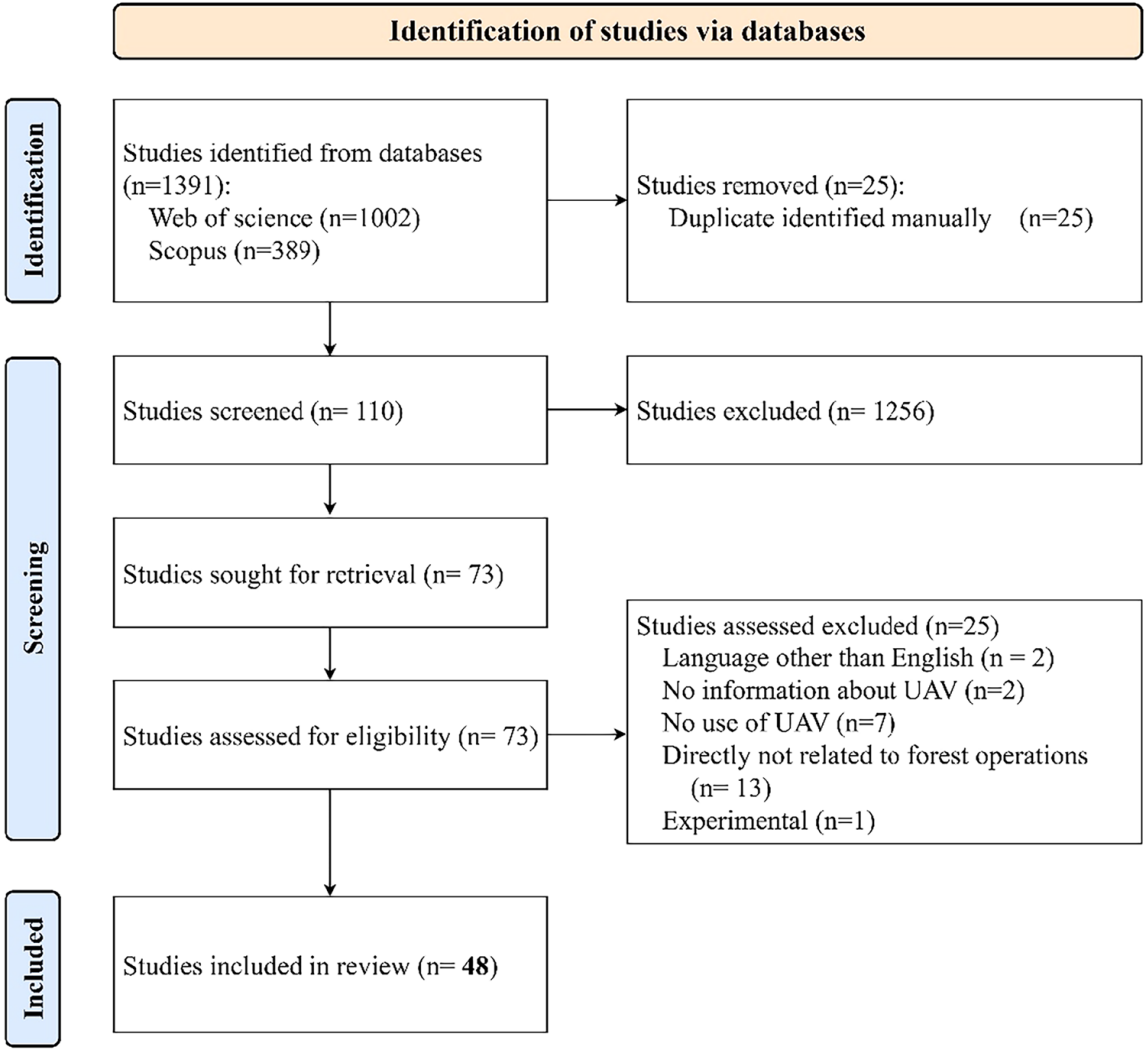
PRISMA flow chart showing the number of publications at different screening stages (n=number of articles)

To collect and analyze the literature from the Web of Science (WoS) and Scopus databases that combine UAVs and forest operations, the Preferred Reporting Items for Systematic Reviews and Meta-Analysis (PRISMA) guidelines of Page et al. (2021) were followed (Fig. 1). The combination of keywords with logical operators; (“drone*” OR “UAV*” OR “unmanned aerial vehicle*” OR “remotely piloted aircraft” OR “RPAS”) AND (“forest operation*” OR “forestry operation*” OR “logging” OR “harvesting” OR “silviculture” OR “felling” OR “skidding” OR “forest road*” OR “forest management” OR “forest inventory”) was applied for a comprehensive search of the relevant literature in both databases in June, 2025. The same Boolean string was applied to both databases. Minor syntactic adaptations required by each platform (TS = in Web of Science; TITLE-ABS-KEY in Scopus) were used. No publication-year filter was applied during the search. The earliest article meeting the inclusion criteria was published in 2016, which defines the natural lower bound of the dataset. Journal research articles were included. To capture the recent emerging work, conference proceedings were also included. However, book chapters and review papers were excluded to avoid duplicating synthesized content and focus on primary data.

The literature datasets were exported as CSV files for further processing. Both CSV files were combined into a single document, and duplicates were manually screened and removed in Microsoft Excel. The references then underwent a multistage screening process consistent with PRISMA guidelines. Both authors independently screened the titles and abstracts of all records. Disagreements regarding inclusion or exclusion were resolved by discussion until consensus was reached. Full-text screening of the remaining articles was conducted jointly, and any further uncertainties were resolved in the same way.

- **Title screening:** We first reviewed the titles of the articles, and any articles deemed off-topic were excluded.
- **Abstract screening:** We read the abstracts of the remaining articles that passed the title screening stage, and off-topic articles were excluded (Ecke et al. 2022).
- **Full-text screening:** The remaining articles that passed the abstract screening were downloaded, and the full texts were read. Articles that did not fall within the scope of forest operations, were in languages other than English, or lacked sufficient methodological or result detail were excluded.

A total of 48 research articles were ultimately considered relevant and included in the analysis with the primary importance of extracting the following information: (1) location of study, (2) objective of the study, (3) UAV model and sensor used in the study, (4) flight parameters, (5) preprocessing and flight planning software used, and (6) key conclusions and limitations regarding UAVs. The extracted information was analyzed via R and presented in the form of figures and tables.

### Selection Criteria

We established specific inclusion and exclusion criteria to ensure the relevance and quality of the review:

- **Inclusion**
  - Studies that have applied UAVs in forest operations such as logging, harvesting, road building, site preparation, and postharvest assessment.
  - Articles published in peer-reviewed journals or conference proceedings.
- **Exclusion**
  - Studies not related to forest operations or UAV-based remote sensing.
  - Non-English language publications.
  - Articles that lack sufficient methodologies or resulting data for synthesis

By applying these criteria during screening, we ensured that the reviewed studies addressed the theme of UAVs in forest operations. We did not apply a formal quality or risk-of-bias tool to the included studies. PRISMA 2020 encourages reporting risk-of-bias methods (Page et al. 2020), but given our objective of describing broad application domains along with trends, limitations, and technical characteristics of UAVs rather than synthesizing effect sizes, we focused on extracting outcomes. This method lines up with recommendations for reviews conducted to contextualize findings rather than evaluate quality by Tricco et al. (2018), Peters et al. (2020).

### Data Synthesis

Forest operation itself is a broad term. For this review, forest operation is divided into five major operational domains or application areas and twelve suboperations. The operational area and sub-operation areas were derived logically from the full-text review of the 48 included articles rather than from a pre-existing typology. Each collected study was assigned to a single topic category according to its primary objective. If a study examined multiple domains, we placed it in the category that best reflected its main aim. In this manner, each study contributes to only one category, avoiding double-counting. Similarly, to provide a visual representation of recurring themes reported in the included articles, we manually reviewed the conclusion or final remarks portion of each study and identified terms that captured the main reported outcomes related to the UAV applications in forest operations. The resulting keyword list was used to generate the word cloud shown in Fig. 5. This figure was intended as a supplementary visual aid to support the narrative synthesis rather than as a standalone analytical result.

## Results

### Overview of Reviewed Studies

**Fig. 2:**
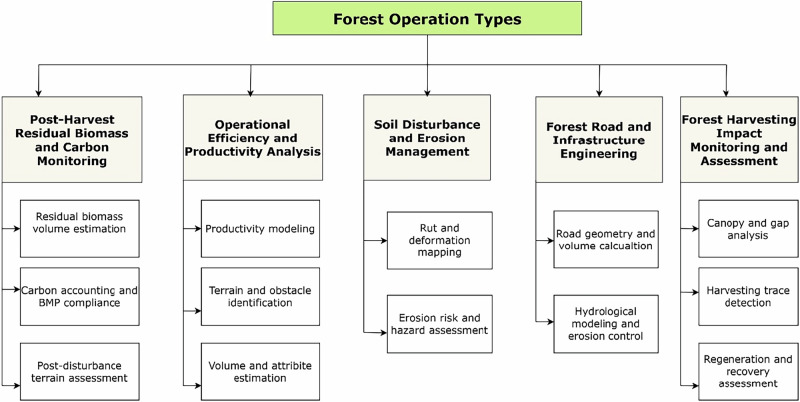
Classification of UAV application areas in forest operations, which are divided into five major domains and their suboperation types

**Fig. 3:**
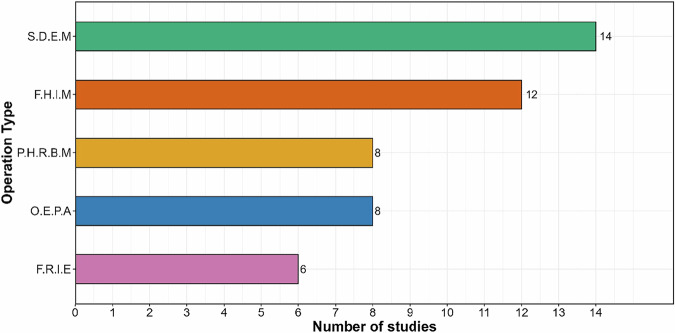
Number of studies on each major operation type

**Fig. 4:**
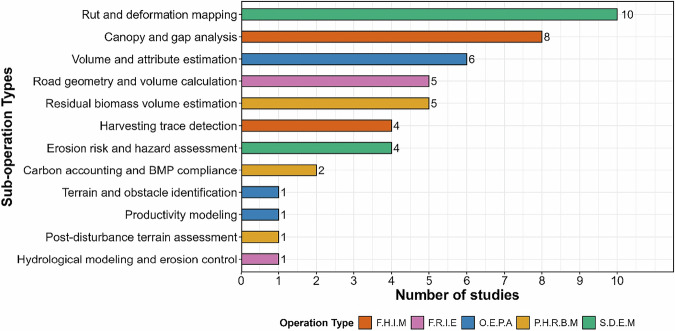
Number of studies in each suboperation type

A total of 48 studies were recorded using UAVs in forest operation activities (see Appendix I for the list of reviewed articles). The forest operation activities span five major forest operation categories: soil disturbance and erosion management (S.D.E.M), post-harvest residual biomass monitoring (P.H.R.B.M), operational efficiency and productivity analysis (O.E.P.A), forest road and infrastructure engineering (F.R.I.E), and forest harvesting impact monitoring (F.H.I.M), which are further divided into 12 suboperation types (Fig. 2). Among the major operation types, the most frequently studied operation type is S.D.E.M (*n* = 14), followed closely by F.H.I.M (*n* = 12). Similarly, the least number of studies published on operation type are P.H.R.B.M (*n* = 8), followed by F.R.I.E (*n* = 6). Below, we present detailed results for each operation category, followed by overall findings, software and flight parameters, limitations, and study trends. (Figs. 3, 4).

### Applications of UAVs in Forest Operations

#### Soil Disturbance and Erosion Management

This is the most frequently studied UAV-based forest operation activity, particularly for rut and deformation mapping, erosion risk, and hazard assessment. UAV-derived imagery, especially from RGB sensors, is constantly utilized to map and determine the depth of wheel ruts and track deformation caused by the movement of heavy machinery (Alvis et al. 2025; Nevalainen et al. 2017; Marra et al. 2021).

Researchers have also integrated artificial intelligence with UAV images for the automatic detection of ruts and erosion modeling. Bhatnagar et al. (2022) and Parajuli et al. (2025) applied deep learning techniques to drone imagery for the automatic detection of wheel ruts and to assess the risk of soil erosion at timber harvesting sites. This integration has improved both detection accuracy and efficiency in analyzing high-resolution UAV data. Similarly, Grube et al. (2025) utilized machine learning technology to measure the rut depth caused by heavy machinery. These studies demonstrate that soil disturbance and erosion management studies have utilized advanced technologies such as deep learning and machine learning.

The utilization of heavy machines on steep slopes hurts the soil and may cause soil erosion. Studies further highlight that UAVs are an effective tool in challenging terrain, such as steep slopes, by assessing rutting and soil compaction caused by forestry machines. UAVs are also reported to be more time and cost-effective than traditional ground-based methods for mapping and detecting skid trails (Latterini et al. 2025; Sealey and Van Rees 2019). In addition to detection, UAVs are being used to classify harvesting tracks into different severities of occurring rut-formation categories, such as light, moderate, and severe, which helps to minimize the environmental effects of heavy machinery (Heppelmann et al. 2022). They can also be used to accurately measure the amount of soil disturbance after a harvesting operation and determine the degree of soil loss (Talbot et al. 2018; Türk et al. 2024). These findings indicate that UAVs can be used as an effective tool for minimizing and monitoring soil erosion in forest operations by identifying high-risk areas before significant degradation occurs. Overall, UAVs have become crucial tools in soil disturbance management by offering intelligent solutions for monitoring machine impacts, mapping sensitive areas, and assisting in formulating mitigation strategies.

#### Forest Harvesting Impact Monitoring

This is the second most studied operation type in which UAVs are used to monitor the impacts of harvesting, which can be further divided into two suboperation types: (a) Canopy and gap analysis, and (b) harvesting trace detection. The UAVs are consistently used for monitoring forest disturbance and logging intensity after logging activities (Aquino et al. 2022; Dupuis et al. 2025). They are flown before and after harvesting to quantify the changes in forest above-ground biomass, stem density, basal area, and canopy cover (Dupuis et al. 2023). Most of these studies have focused on detecting the impacts of selective logging. These UAV-based assessments outperform the traditional methods and satellite imagery in spatial resolution. Various studies have integrated UAV imagery with deep learning and machine learning technologies to automate canopy gap detection and identify information about tree stumps, such as size and volume (Htun et al. 2024; Kamarulzaman et al. 2022).

In trace detection, UAVs are consistently used for mapping and estimating the size, area, and number of stumps and detecting felled trees (Samiappan et al. 2017; Thiel et al. 2020). This information can be used to identify the logging intensity. Also, mapping stumps and felled trees will assist in the management of post-harvest activities and ensure sustainable harvesting practices. UAVs derived datasets are being used as reference data for comparison of accuracy between satellite imagery, airborne LiDAR, and field measurements (Castillo et al. 2022; D’Oliveira et al. 2021). These studies confirm that the UAV-imagery can be used as validation data and show the superior granularity of UAV data.

Overall, these findings illustrate that UAVs are increasingly used to monitor the impacts of logging and provide information on canopy gaps. We found that most of these works focused on selective logging. UAV imagery provides practical information to enforce logging regulations by identifying the extent and intensity of logging activities.

#### Operational Efficiency and Productivity Analysis

UAVs are consistently used as an essential tool for enhancing operational efficiency and productivity analysis in forest operations. Eight studies in our systematic review addressed this area, focusing primarily on (a) volume estimation, (b) productivity modeling, and (c) terrain and obstacle identification for operational planning.

We found that UAVs can be efficiently used for estimating the volume of timber and timber-derived products, such as wood chips. Mokros et al. (2016) reported that UAVs are time-effective when collecting data for the estimation of volume. Similarly, the use of UAVs can outperform some traditional methods and some terrestrial laser systems, such as laser satellites, mobile laser scanners, smartphones with LiDAR, and TLSs, in terms of both temporal and economic points of view for volume calculation (Gejdos et al. 2024; Duka et al. 2023). Interestingly, we did not find any studies that used UAVs to calculate the roundwood pile volume in forest mills, suggesting a potential area for future work.

Machine learning techniques are applied with UAV imagery to estimate the weight of wood and the residual volume of stands (López-Amoedo et al. 2023; Goodbody et al. 2017), proving UAVs as automated inventory tools. Furthermore, some of the studies suggest that the UAVs can be used as a real-time operational monitoring tool (Hou et al. 2022). From a productivity modeling perspective, UAVs have been used to perform time-motion studies of heavy machinery, such as a forwarder, to assess the productivity and inefficiencies (Nunes et al. 2021). UAVs offer a unique advantage for observing machine cycles over large forest areas, although their limited battery life remains an operational challenge.

Terrain analysis for operational planning can be performed effectively via UAVs. Timber extraction is an important and difficult step, especially if timber harvesting is performed on rugged terrain. The skid trails may have different obstacles, which may slow the timber extraction process. UAV-derived images can be used to identify such types of obstacles and assist in planning the logging-transport process (Hruza et al. 2025). Overall, studies of this operation type indicate that UAVs are emerging as multipurpose tools for volume assessment, operational monitoring, and terrain analysis, offering both tactical and strategic support for improving operational efficiency and productivity analysis.

#### Post-harvest Residual Biomass and Carbon Monitoring

This is another important application area for UAVs, which includes (a) residual biomass volume estimation, (b) carbon accounting and BMP compliance, and (c) post-disturbance terrain assessment. These applications are crucial for sustainable forest management and environmental compliance. Various studies have found that UAVs are consistently used to calculate the volume of forest residue piles, such as coarse woody debris, and assess the spatial distribution and classification of such residues across the harvested area (Harvey 2022; Sealey and Van Rees 2020). Recent advancements in technology have further integrated machine learning techniques for automatic detection and classification of such residues (Udali et al. 2022; Windrim et al. 2019).

Another emerging application is UAV-assisted monitoring of Best Management Practices (BMPS). Rijal et al. (2023) used UAVs to evaluate the feasibility and potential of UAVs in BMP monitoring, and they reported that UAVs can be effective tools for that. This finding indicates that UAVs can assist in ensuring environmental compliance after harvesting.

In terms of carbon accounting, UAVs can be used to quantify carbon emissions after selective logging. For example, Saad et al. (2023) utilized a UAV survey to assess the impact of selective logging by quantifying carbon emissions from selective logging activities. UAV-based imagery can be utilized to create digital terrain maps (DTMs) of sites after a disturbance to an area, such as a disaster or wildfire. Duka et al. (2022) utilized UAVs to make DTMs in salvage logging. These UAV-derived terrain maps can reveal changes in the sites resulting from both disturbances and the subsequent logging activity. These DTMs are useful for planning site preparation or reforestation efforts. In summary, UAVs are proven to be powerful tools for precise automation in assessing biomass and carbon dynamics, monitoring the management practices after post- logging activities.

#### Forest Road and Infrastructure Engineering

With respect to this type of operation, the fewest studies have been conducted thus far, but it is one of the most critical aspects of forestry operations. To cut, skid, and haul a tree, the basic requirements are forest roads and skid trails. UAVs are being employed for forest road surveys, design, and monitoring activities. UAVs have proven to be an effective tool for the estimation of the volume of the forest road cut (Türk et al. 2024) and to prepare high-definition maps for road monitoring and planning for geometry and road conditions (Cheng et al. 2023). These UAV-derived datasets are more efficient than ground-based methods.

UAV imagery can be used for collecting precise profile data for evaluating road grade compliance. For instance, Lepoglavec et al. (2023) used UAV imagery to determine the longitudinal slope of an existing skid trail. From an infrastructural engineering point of view, UAVs can be used to record normal cross-sections, side ditch depths, and culvert dimensions on forest roads (Papa et al. 2025; Açil et al. 2023). In summary, although the application of UAVs is underrepresented in literature, they hold significant promise in forest road engineering by providing cost-effective, high-resolution monitoring of infrastructure, which is crucial from a safety perspective.

### Synthesis of Key Findings

**Fig. 5:**
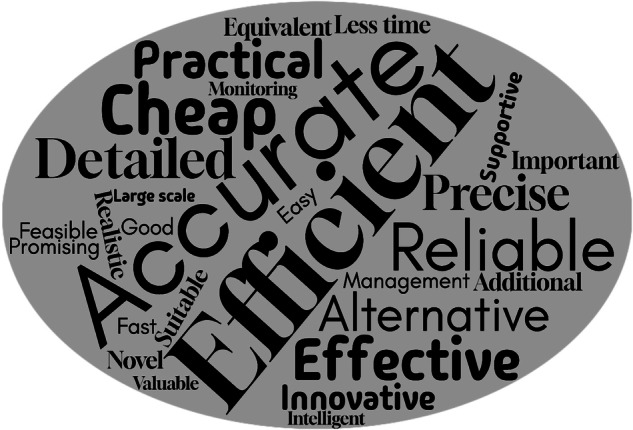
Word cloud summarizing recurring terms related to the use of UAVs in forest operations. Larger words indicate more frequent mention in the conclusion or final-remarks sections of the reviewed studies. Key findings regarding the use of UAVs in forest operations. Larger words indicate greater occurrence in the conclusions of the reviewed studies

Figure 5 provides a visual summary of the recurring terms identified from the conclusion or final-remarks sections of the studies included. The most frequently represented terms reflected positive perceptions of UAVs, particularly their efficiency, accuracy, practicality, reliability, and cost-effectiveness.

### UAV Platforms and Sensor Types

#### UAV Platforms

**Fig. 6:**
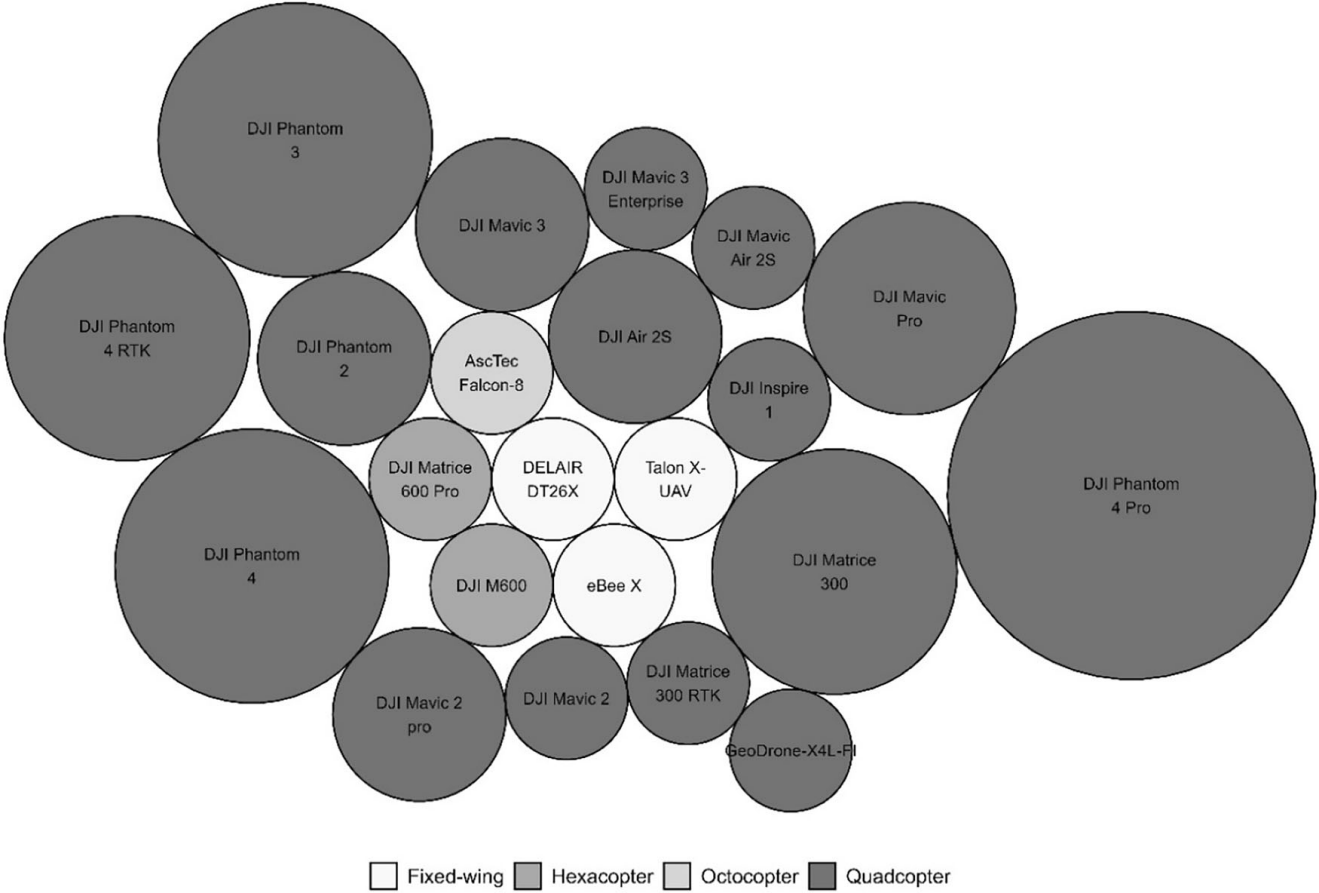
UAV model (circles sized by frequency of use)-quadcopter-like DJI Phantom dominating

Most of the studies used multirotor UAVs, particularly quadcopter models, rather than fixed-wing UAVs. Figure 6 shows the distributions of the specific UAV models used. The most used platform is the DJI Phantom 4 Pro quadcopter, followed by other models such as Matrice, Mavic, and Inspire series. We believe that the dominance of multirotors is likely because they have greater maneuverability and ability to hover, which makes them good tools for mapping small areas, skid trails, wood chip piles, and wood yards. Fixed-wing UAVs, on the other hand, generally cover larger areas but are used very little in these studies. Compared with fixed-wing UAVs, multirotors are less expensive and simpler to operate in rugged terrain.

#### Sensor Types

**Fig. 7:**
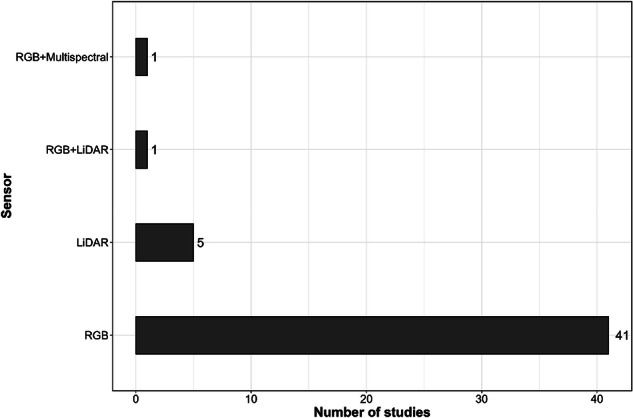
Distribution of sensor types used-RGB cameras-in nearly all studies, with a few using LiDAR and multispectral sensors

The integrated sensors carried out by UAVs in these studies were dominated by RGB sensors. Figure 7 shows the distribution of different types of sensors attached to UAVs in forest operations. Approximately 85% of the studies reported the use of RGB cameras. A few studies have combined RGB with other sensors, such as multispectral and LiDAR. In particular, the use of LiDAR has increased significantly since 2022, with the highest number of LiDAR-equipped studies reported in 2025. LiDAR sensors were used in only 5 studies, mainly for monitoring forest disturbance after selective logging, for acquiring 3D forest attributes, weight estimation of wood, identifying surface deformation, and mapping forest trails. It is combined with an RGB to identify terrain obstacles encountered during timber extraction in the skidding process. Similarly, a multispectral sensor was used occasionally in combination with an RGB camera to examine the impact of the skidder on the soil and regeneration. We found no use of a hyperspectral sensor in forest operations, and it may be because of the cost and complexity. Overall, we noted the preference for a cost-effective and high-resolution RGB and the emerging use of LiDAR capable of performing 3D measurements (Fig. 7).

### Software Tool and Flight Parameters

#### Image Processing and Analysis Software

**Fig. 8:**
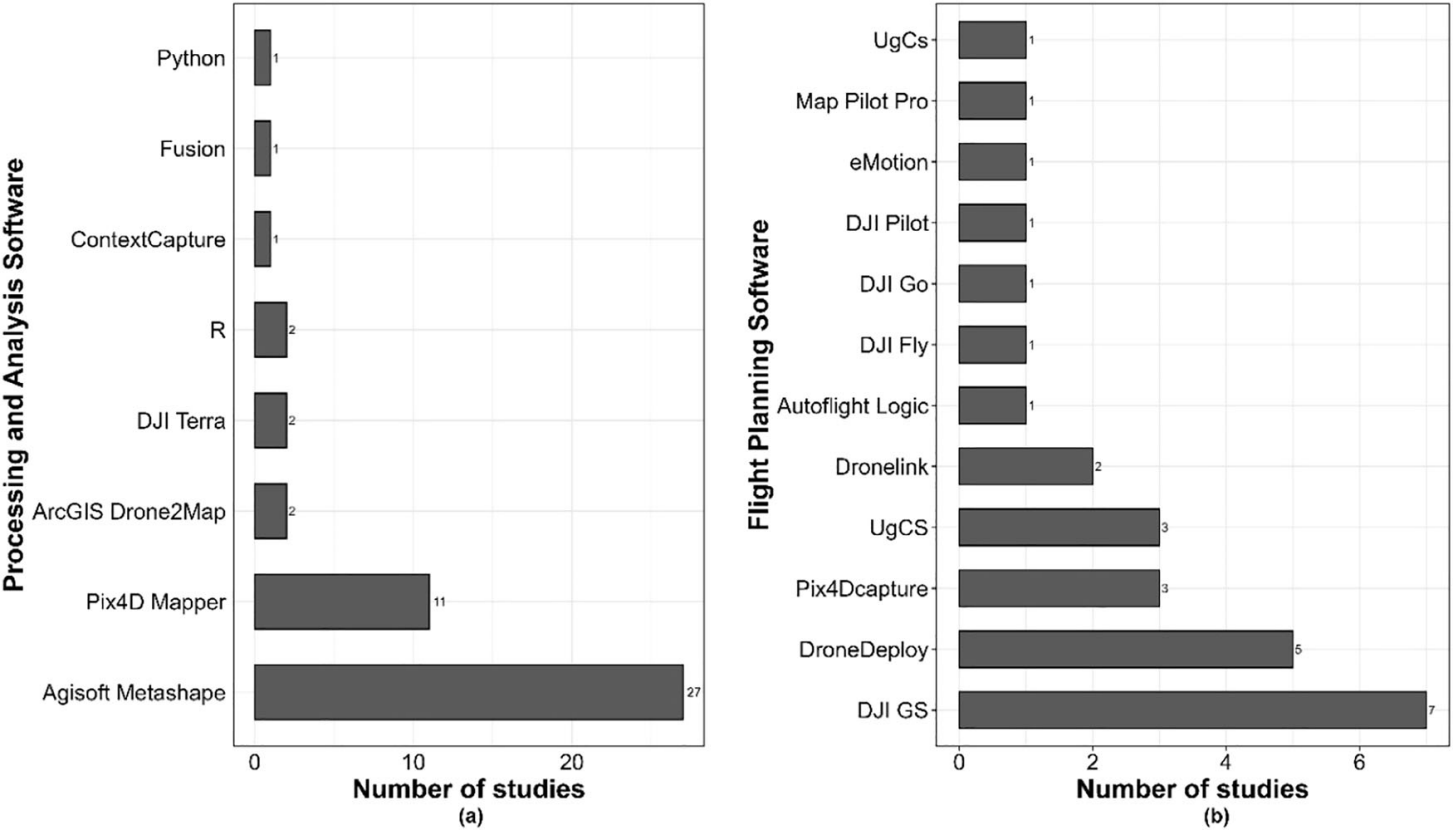
**a** Preprocessing and analysis software. **b** Flight planning software

Figure 8a summarizes the processing and analysis tools used for processing UAV imagery and data analysis. Most of the studies ( ~ 56%) used Agisoft Metashape for photogrammetric processing of images (creating DEMs and orthomosaics). Pix4D Mapper was the second most common preprocessing software used ( ~ 23% of the studies utilized it). This software provides a user-friendly workflow for processing drone imagery. After preprocessing, some of the studies utilized statistical or GIS software for further analysis. For example, R was used for detecting skid trails, and fusion (a LiDAR analysis software) appeared when handling point clouds. Python was also used along with R in the same study.

#### Flight Planning Software

Figure 8b shows the software tools used to plan UAV flights. The most utilized software was the DJI ground station (GS). It was reported to be used in 7 studies. This reflects the heavy use of DJI drones, which integrate with DJI’s own flight planning software. Similarly, DroneDeploy was the second most utilized flight planning software. Pix4D capture, which was reported in 3 studies, is also used by some users for automatic flight planning. These software tools allow users to set flight parameters such as flight height, front and side image overlaps, and velocity to ensure adequate coverage and image quality. However, most of the studies have not mentioned the flight planning software they have used.

#### Flying Height

**Fig. 9:**
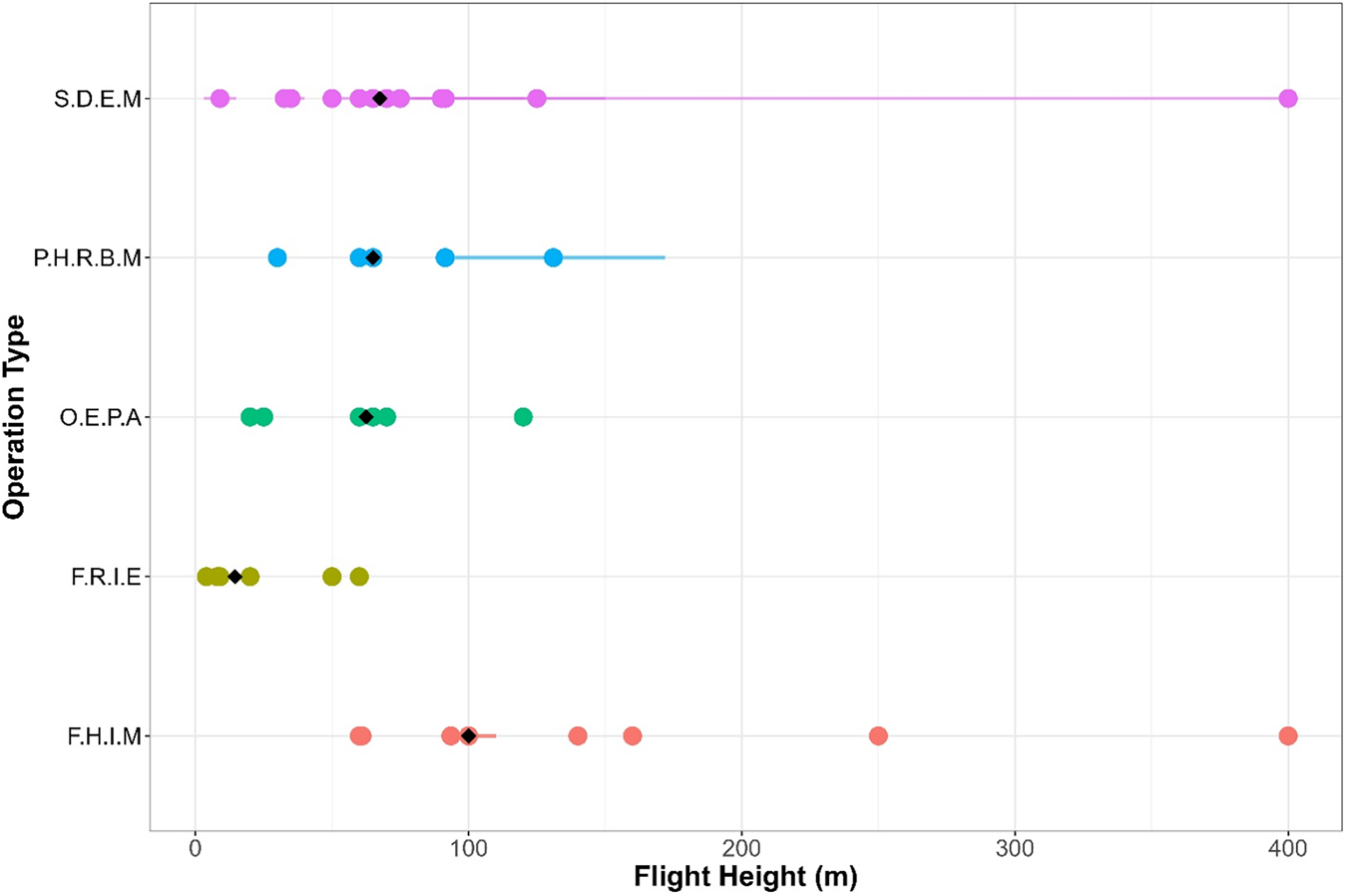
UAV flight altitude by operation type, with higher flight altitudes for harvest impact monitoring and lower for forest road surveys. Each colored point represents the exact flight height/average flight height reported in the literature. The horizontal lines indicate the full range of flight altitudes (minimum to maximum) for studies that reported intervals, and the black diamonds denote the median flight height for each operation type. The operation type is abbreviated as: F.H.I.M. Forest Harvesting Impact Monitoring and Assessment, F.R.I.E. Forest Road and Infrastructure Engineering, O.E.P.A. Operational Efficiency and Productivity Analysis, P.H.R.B.M. Post-Harvest Residual Biomass and Carbon Monitoring, and S.D.E.M. Soil Disturbance and Erosion Management

Figure 9 shows the range of UAV flight altitudes used across different operation types. The flight altitude significantly influences image coverage and resolution. While data extraction, we found that some of the studies reported flight altitude within a range (e.g., 60–120 m), and some of them reported exact altitudes. For the range, we computed the mean altitude and showed both the range and the mean altitude. The flight altitudes used in UAV surveys differ according to the objectives and scale of the operation type. For example, studies in forest road engineering tend to focus on relatively lower altitudes ( ~ 15–30 m) to capture fine details such as road excavation volumes and hydraulic structure dimensions. On the other hand, forest harvesting impact monitoring frequently uses relatively high altitudes ( ~ 100 m, median height) to cover large areas. The soil disturbance and erosion management operation type has a median flying height of 60–70 m, which is the same for postharvest residual biomass monitoring, operational efficiency, and productivity analysis (Fig. 9). Even within the same operation type, the flight altitude varies widely depending on the objective, e.g., some harvest impact monitoring studies have used lower altitudes (for harvesting trace detection), and some have used higher altitudes (for monitoring canopy disturbances after selective logging).

#### Image Overlap

**Fig. 10:**
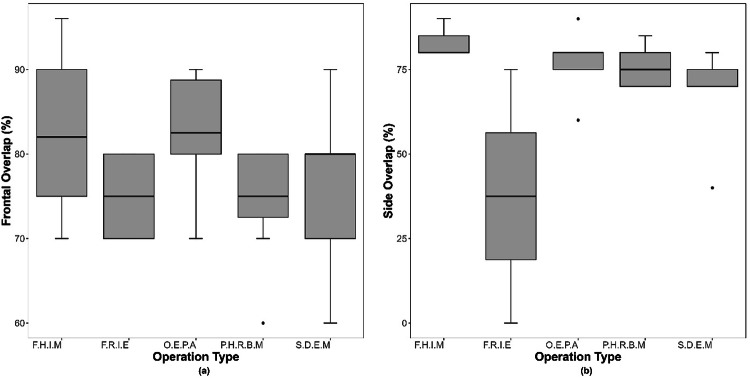
Front and side overlap in UAVs according to the operation type. Operation Type abbreviations: F.H.I.M.: Forest Harvesting Impact Monitoring, F.R.I.E.: Forest Road and Infrastructure Engineering, O.E.P.A: Operational Efficiency and Productivity Analysis, P.H.R.B.M: Post-Harvest Residual Biomass Mapping, and S.D.E.M: Soil Disturbance and Erosion Management

Figure 10 shows a boxplot of the distributions of (a) frontal overlap and (b) side overlap (%) settings used in UAV-based forest operations across different forest operation domains. These overlaps are important for successful photogrammetry.

- Frontal Overlap (%): Frontal overlap values ranged widely across operation types, with F.H.I.M., O.E.P.A., and S.D.E.M. showing higher median values (~80-85%), indicating a preference for denser forward image capture. In contrast, F.R.I.E. and P.H.R.B.M. exhibit lower medians and narrower interquartile ranges, suggesting more moderate overlap practices. F.H.I.M. has a wider range of front overlaps from 75% to 90% with a median of 83%, which is the same as the median of O.E.P., but the range of overlaps there is quite small. Similarly, the range of front overlaps of the F.R.I.E. and P.H.R.B.M. is almost the same, with an exact median of approximately 75%. In S.D.E.M., the third quartile and median number are equal at 80%, with Q1 at 70%.
- Side Overlap (%): Side overlap varied more dramatically. Most operation types, such as O.E.P.A, P.H.R.B.M., and S.D.E.M., showed consistently high side overlap ( ~ 70-80%). However, F.R.I.E. has a wider distribution with lower values (median ~40%). F.H.I.M. has

These results suggest that the overlap settings depend upon the operation type.

### Challenges and Limitations

UAVs have been proven to be accurate and alternative methods in forest operations; however, they have limitations. The major challenge with UAVs is that they are prone to obstacles such as branches and leaves (Hasegawa et al. 2023), making it difficult to operate under the canopy (Açil et al. 2023). More specifically, we found that RGB sensors were prone to obstacles. In addition, the studies stating UAVs are prone to obstacles include applications such as forest road cut volume estimation, forest road hydraulic structure dimension estimation, and forest road map generation. Therefore, the accuracy of models generated via UAV imagery cannot be guaranteed (Cheng et al. 2023). Another major limitation for UAVs is their limited battery life (Lepoglavec et al. 2023). The average flight time of most UAVs is ~30 minutes per battery.

For UAV operations, trained and specialized personnel are required to operate the UAVs (Windrim et al. 2019). The use of UAVs depends upon the objective of the study, yet it can be costly. Gejdos et al. (2024) used different methods for data collection and analysis, including UAVs, and reported that processing UAVs is difficult and time-consuming compared with other tools. Additionally, UAVs are limited to small areas (Dupuis et al. 2023). If the objective is to map a large area, we must use satellite imagery or take imagery from manned aircraft. To obtain accurate results, the use of GCPs is recommended. GCPs are permanent marks of interest that help with base referencing. The installation and surveying of GCPs is time-consuming (Thiel et al. 2020). UAVs have been widely used to estimate wheel rut depth. When the ruts are covered with brush mats, filled with water, or with many residuals, UAVs cannot accurately estimate the depth of the ruts (Marra et al. 2021; Kim et al. 2025).

In summary, the main limitations of UAVs in forest operations include data processing time, limited coverage per flight, and flight constraints such as obstacles and battery range. These factors currently restrict some operational uses of UAVs, but many of them can be mitigated.

### Publication Trends

**Fig. 11:**
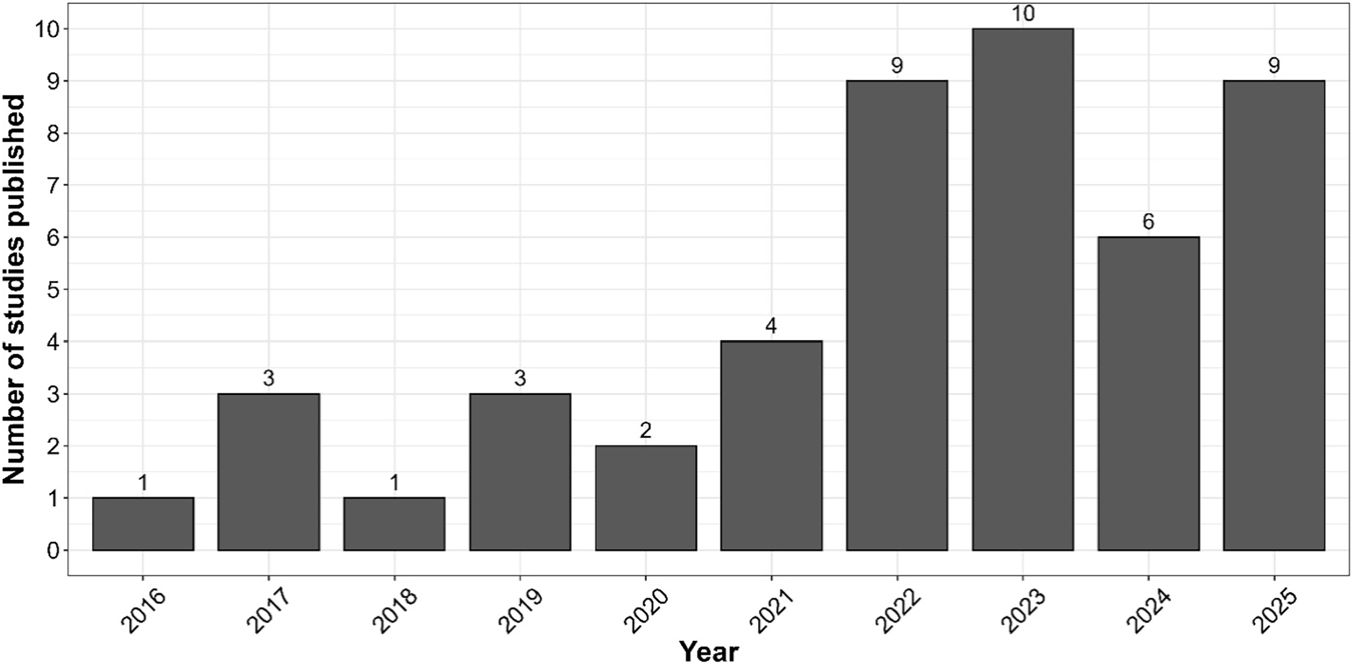
Year-wise publication of studies utilizing UAVs in forest operations

The use of UAVs in forest operations has significantly increased over the past few years. Figure 11 shows the number of studies published each year using UAVs in forest operations. The number of publications significantly increased from 2020 onward, with the highest number of studies occurring in 2023 (*n* = 10), whereas 2016 and 2018 received a smaller number of studies (*n* = 1). The preliminary data for 2025 suggest that the high rate is continuing. This trend shows a growing interest in the use of advanced technologies. The use of advanced sensors such as LiDAR has significantly increased since 2022, with the highest use reported in 2025. Similarly, the integration of AI and deep learning in analyzing UAV data has been reported since 2020. In short, we conclude that the 2020 -2025 period represents a time of rapid expansion and technological advancements in UAV-based forest operations studies.

### Geographic Distribution

**Fig. 12:**
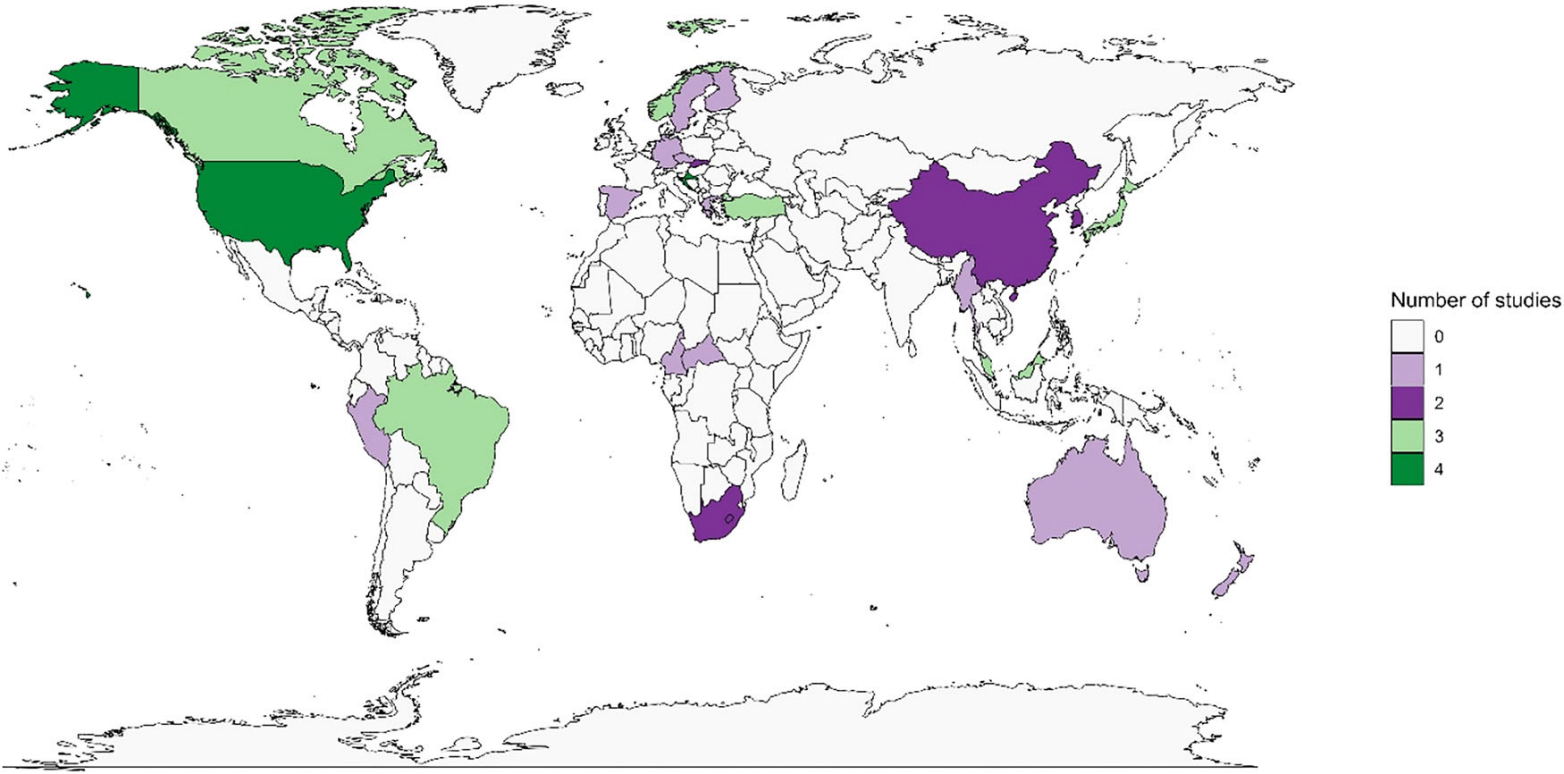
Geographical distribution of studies using UAVs in forest operations. Darker shading = more studies

Studies on UAVs in forest operations have a broad global scope. Figure 12 illustrates the utilization of UAVs in forest operations by country. The United States and Croatia are the countries publishing the greatest number of studies (*n* = 4), followed by Turkey, Norway, Malaysia, Japan, Canada, and Brazil (*n* = 3). The strong representation of the USA is expected, given its large forestry sector and research capacity, whereas an active research community focusing on forest engineering has resulted in a greater number of studies in Croatia. In total, every continent is represented in this review, which shows a global interest in UAV-based forest operations.

Most of the studies are reported from North America (the USA and Canada) and Europe (leading to Croatia and Norway). On the other hand, fewer studies have been conducted in Africa or South America outside of Brazil. We also observed that most of the LiDAR-equipped studies in Europe and Asia reflect newer technologies in those areas.

Overall, geographic analysis suggests that there is widespread use of UAVs in forest operations, not limited to any single region. Developed countries with advanced forest industries are leading the research, but emerging economies are also exploring the use of UAVs.

### Publishers and Journals

**Fig. 13:**
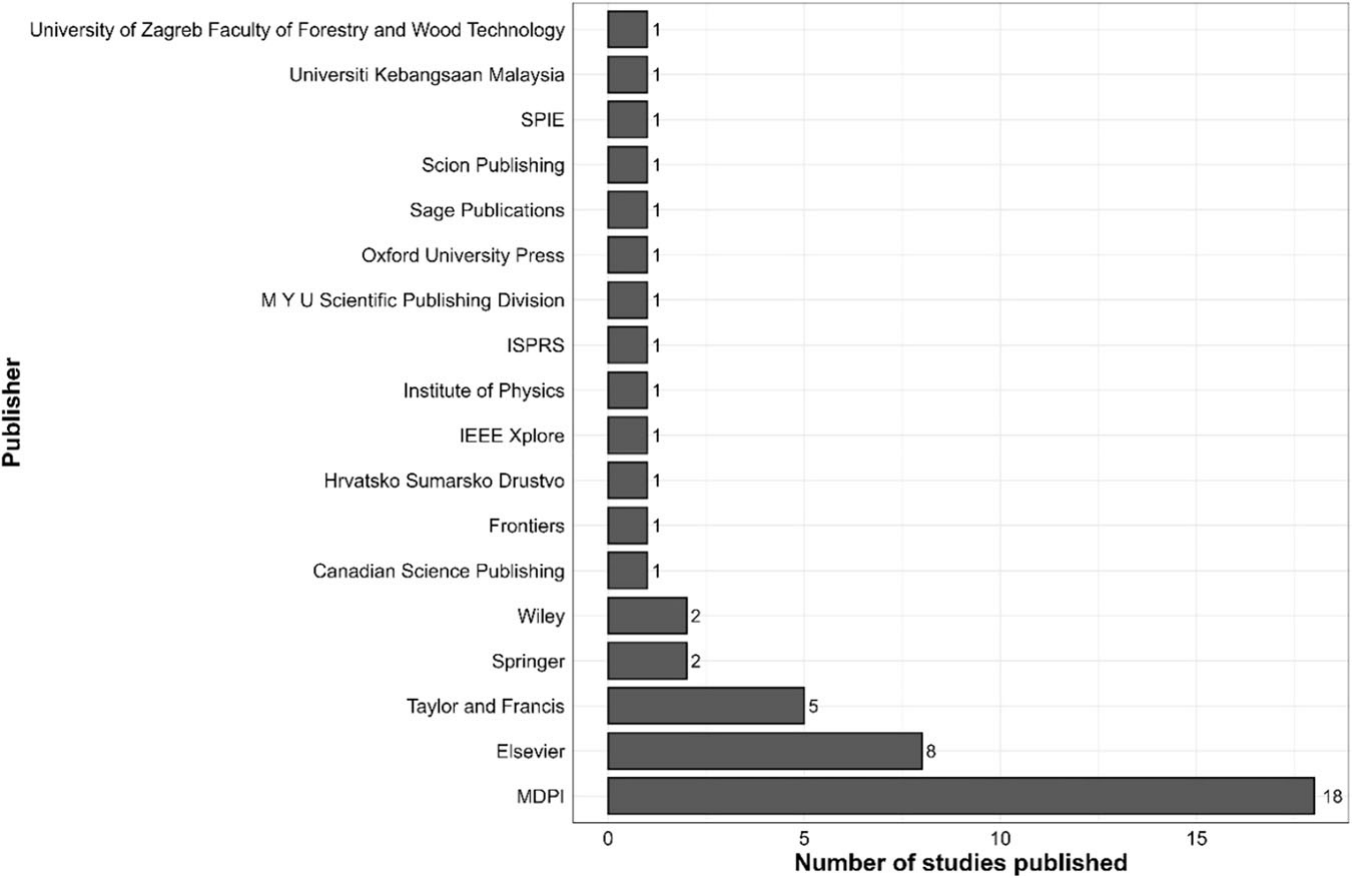
Distribution of studies by publishers. The MDPI published the largest number of studies utilizing UAVs in forest operations

The studies we reviewed were published through a variety of journals and conference proceedings. Figure 13 shows the distribution of studies by publishers. The Multidisciplinary Digital Publishing Institute (MDPI) published the greatest number of studies (n = 18 out of 48). Forests, Drones, and Remote Sensing were the most common journals under MDPI. The next most common publisher was Elsevier, which published 8 studies in journals such as Forest Ecology and Management, Remote Sensing of Environment, Trees, Forests and People.

We found that thirteen (13) publishers published at least one (1) study on UAVs in forest operations. Springer, Taylor and Francis, and various conference proceedings (IEEE Xplore, ISPRS) were other notable publishers in addition to MDPI and Elsevier. These studies have received much attention from publishers. The presence of multiple journals reflects a trend toward the wider dissemination of results to researchers and academia. This indicates that the use of UAVs in forest operations is receiving considerable attention and recognition as an emerging field of importance. However, the broad academic publication does not necessarily mean interpreted as adopted by practitioners who rely on extension publications, public presentation, and technical bulletins; depicting practitioner-facing dissemination would require a separate review of the gray literature.

## Discussion

These reviews focused on the use of artificial intelligence with UAV imagery. Machine learning and deep learning techniques, which are considered advanced technologies, have been employed. Most of the studies have applied a machine learning algorithm along with UAV imagery. This systematic review revealed that UAVs are rapidly becoming an important tool in multiple forest operation domains. This review identified 5 major operational domains. Among them, the most frequent application areas are soil disturbance and erosion management, followed by forest harvest impact monitoring. Within these application areas, UAVs have been utilized to map rut sand deformation caused by machinery, measure soil erosion risk, identify canopy gaps, perform trace detection, and assess regeneration. The least utilized fields were forest roads and infrastructure, which involve determining the forest road excavation volumes, measuring the dimensions of hydraulic structures on forest roads, preparing forest road maps, and determining the slopes of forest roads. Although few studies exist, those studies identified UAVs as alternative methods that provide good accuracy with reliable results. This shows that despite the lower number of studies, the results are promising. Most of these studies reported that UAVs are prone to obstacles, and we assume that this might be one of the reasons for fewer studies utilizing UAVs in this application area. More studies should be conducted on forest roads and infrastructure engineering utilizing LiDAR sensors, which can penetrate dense canopies. The same results were obtained from postharvest residual biomass monitoring. This field has been the subject of fewer studies, but the utilization of UAVs has proven to be an alternative and effective method for determining residual slash volume. This could become especially relevant under climate-oriented forest management, where accounting for residual biomass and carbon emissions is crucial.

This review has shown that by using UAVs, efficiency and accuracy are greater than those of conventional methods. Many studies have reported that UAV-based measurements (slash volume determination and wood chip pile volume estimation) are not only faster and less expensive but also more accurate than existing ground-based methods (Gejdos et al. 2024; Windrim et al. 2019). In some cases, UAV imagery is used to assess the accuracy of Landsat-8 and Sentinel-2 imagery. There are, of course, activities where UAVs are less practical. For example, Dupuis et al. (2023) reported that UAVs are not ideal for larger areas, and still omit some small gaps; however, the technology is continuously improving. One noticeable gap identified is roundwood pile volume estimation in the forest mill; while UAVs have been used for chip piles and felled stems, we did not find studies on roundwood pile volume estimation. This could be a valuable operational application area, which is considered an issue. Further research needs to be done in this field.

We found that most of the studies did not report detailed flight parameters such as flying height, front and side overlaps, speed, etc. This is a significant issue, as recent studies show that these parameters substantially affect the quality of data. For instance, Dhruva et al. (2024) suggested optimal UAV photogrammetric settings as 90% and 85% front and side overlaps, respectively, at an altitude of 120 m for forest surveys. Similarly, Eisenschink et al. (2025) reported that differences in flight parameters have a great effect on the accuracy of the data we want to collect. They also recommend flying UAVs at low altitude, at relatively high speed, with high overlaps. Importantly, optimal flight parameters vary by application type. We recommend that future studies record and report flight parameters consistently to improve reproducibility.

The geographic distribution of studies utilizing UAVs in forest operations suggests that interest in UAVs for forest operations is widespread. Developed countries such as the USA and Croatia, with active forest industries, have led early research, but we observed adoption in tropical regions such as Brazil and emerging economies as well. This indicates that UAV technology applies to various forest types and management contexts, from industrial plantations to selective logging. We believe that the reason behind this broad adoption of UAVs in forest operations is the decreasing cost and increased usability of commercial UAVs. By the early 2020 s, many forestry researchers and companies could afford high-quality UAVs at a lower cost, which likely contributed to the increase in the number of publications we observed after 2020.

Another notable observation is the rapid integration with technological advancements in this field. Since 2020, a significant amount of research has incorporated AI, particularly machine learning and deep learning, with UAV-based remote sensing to improve forest operations. Ten (10) of the 48 studies applied ML/DL algorithm across diverse operational areas, including wheel-rut detection, felled tree volume estimation, soil disturbance classification, canopy gap analysis, coarse woody debris and stump detection, and forest road cut volume estimation (Table 1). Table 1 summarizes the performance of the most used deep learning and machine learning models, including random forest, SVM, regression algorithms, and CNNs, across a range of forest operations activities. The Random Forest models, for instance, in logging residue mapping and road-volume estimation tasks, achieved a high classification accuracy of around 89% with Cohen’s k ranging from 0.83 to 0.86 (Udali et al. 2024; Türk et al. 2024). Similarly, it has achieved predictive R^2^ in the range ~0.69–0.85 for regression tasks like predicting rut depths (Grube et al. 2025).

**Table 1:** Summary of model performance for ML/DL algorithms

Model type	Task/Study	Metrics	Performance (Key results)
Random Forest	Logging residue classification (Udali et al. 2024)	Overall accuracy, Kappa	Overall accuracy = 0.89; Kappa = 0.83
	Forest road cut volume estimation (Türk & Canyurt. 2024)	Overall accuracy, Kappa	Overall accuracy = 89.09%; Kappa = 0.86
	Rut depth prediction on peatland trails (Grube et al. 2025)	R² (regression fit)	Predictive R² = 0.69–0.85
Support Vector Machine	Post-logging carbon emission modeling (Saad et al. 2023), canopy gap detection (Kamarulzaman et al. 2022)	RMSE, Bias, Adjusted R², overall accuracy	RMSE 21.10%, bias 0.23%, R² = 0.80, overall accuracy = 0.85
Regression models	Felled tree volume estimation (Hou et al. 2022)	R², RMSE	Best model: R² = 0.9796; RMSE = 0.0584
CNN (Deep Learning)	Wheel rut detection in harvested sites (Bhatnagar et al. 2022)	F1-score, detection accuracy	F1-score 0.69–0.84 (avg 0.77); ruts detection accuracy = 79%
	Soil disturbance classification (Parajuli et al. 2025)	Precision, Recall, F1-score	Best model: DeepLabV3 + ResNet-34 (F1 ranged 0.902–0.985 across classes)
	Canopy gap analysis (Htun et al. 2024)	F1-score, OA, IoU	Best model: ResU-Net_2 (F1 = 0.56, OA = 0.83, IoU=0.39)
	woody debris and stump detection (Windrim et al. 2019)	Precision, recall, R²	precision=83.9%, recall=81.8%, R^2^ = 0.95

For carbon emission modeling using SVM, the best model achieved an RMSE of ~21.1% with a very low bias (0.23%) and an adjusted R² of 0.80, indicating a strong fit. Similarly, while estimating tree volumes from UAV, a regression-based volume model achieved a near-perfect fit with R^2^ ~ 0.98 and RMSE ~ 0.0584. These results indicate that machine learning models can achieve high accuracy. CNN/deep learning models have reported metrics such as F1 score, which ranges from 0 to 1, where 1 indicates perfect prediction, and accuracy. One convolutional model used for wheel rut detection has achieved an F1-score ranging from 0.69 to 0.84 and a detection accuracy of 79%, while a DeepLabV3 network for soil disturbance classification reported an F1 score ranging from 0.902 to 0.985 across classes (Bhatnagar et al. 2022; Parajuli et al. 2025). For automatic canopy gap detection, both machine learning and deep learning algorithm is applied, and it has achieved an overall accuracy of about 83-85%. Similarly, deep learning models has shown convincing results for stump detection, coarse woody debris mapping and volume estimation (Windrim et al. 2019). These results highlight that utilizing deep learning with UAV imagery has achieved more accuracy in prediction. Overall, forest operations utilize advanced technologies for handling large datasets and extracting complex patterns. Although the number of studies is limited, we expect enhancements in the use of AI for increased accuracy and automation in identifying features such as individual logs, damage detection or erosion features from UAV imagery, finally transforming decision-making in forest operations.

Although the overall results are positive, it is important to recognize and address the limitations and challenges of UAV technology. Many studies highlight practical issues such as limited flight time, small area coverage, longer processing times, and the time-consuming process of installing GCPs. In terms of a small coverage area, Bakirci (2024) uses a distributed UAV network: the area is divided into sub-areas by UAV capability, enabling each UAV to cover its region and map pollution consistently. This approach can be used in forests with large areas to map the operational tasks. For operational use, the forest industry and related organizations should consider implementing training programs to help personnel operate UAVs effectively, ensuring safety and maintaining data quality. Most of the synthesized studies are academic; very few of these mentioned works are being deployed in industry. One example is the volume estimation of wood chip piles. Additionally, most of the work done in industry utilizing UAVs is not published in scientific journals. We identify this as a gap between academia and industry. We recommend that future research involve surveys of forest industries to explore how they use UAVs in the field and the challenges they face. A potential drawback of this review is the exclusion of non-English studies; during screening, we identified two potentially relevant studies that were excluded for this reason. The geographic distribution reported in section 3.8 should therefore be interpreted as the distribution of English-language UAV-in-forest operations research rather than as the true global distribution of UAV adoption. Similarly, this review was limited to peer-reviewed studies indexed in Scopus and Web of Science; gray literature such as theses and technical reports was excluded. It may have introduced publication bias by potentially missing relevant unpublished or non-indexed findings.

## Future Directions

This review suggests several future directions and opportunities for UAVs in forest operations. One of them is the use of LiDAR and other sensors. Most studies have reported that UAVs are prone to obstacles such as leaves and canopy cover. The majority of these methods are based on forest road and infrastructure operations, such as forest road cut volume estimation (Hasegawa et al. 2023), road hydraulic dimension estimation (Açil et al. 2023), and forest map preparation (Cheng et al. 2023). A LiDAR sensor can penetrate through the gaps between the leaves, and some of the information can be obtained (Wagner et al. 2008). However, the use of LiDAR was minimal. The UAVs equipped with cost-effective LiDAR sensors would be helpful for limiting these challenges. Similarly, thermal and multispectral sensors can be used to determine the soil moisture variation in skid trails (Bertalan et al. 2022), detecting hidden stump decay that was not explored in these reviewed studies.

Technological advancements can minimize the current limitations of UAVs. Some studies find that UAVs are prone to obstacles and have limited battery life. Recent advancements have introduced obstacle avoidance systems, which help reduce the risk of crashes. The production of batteries with longer flight times should be created. Similarly, the longer time for GCP installation is minimized when RTK UAVs are used, but they are more costly than other UAVs. Therefore, these factors need to be considered.

We also expect UAVs to provide real-time operational monitoring of the woods being harvested. Currently, UAVs are used after post-harvesting activities to obtain results. However, in the future, UAVs can be deployed during operations. For example, UAVs could follow a feller buncher or a harvester to obtain real-time data on the amount of wood harvested or the area of the forest block cleared, which helps in the better decision-making process. It will require onboard computation or rapid transfer of data combined with strong AI to interpret the data during the flight.

## Conclusion

This systematic review synthesized 48 studies to demonstrate the use of UAVs in forest operations. We found that UAVs are used in multiple aspects of forest operations to plan and monitor harvesting, assess forest road conditions, and evaluate soil disturbance, productivity modeling, and other operational impacts. Across these operational fields, UAVs are efficient, reliable, precise, cheaper, and provide accurate, more detailed data than conventional methods do. We also identified multirotor and RGB sensors as the most common drone platforms and sensors, and their use has spread globally and has rapidly increased in recent years. For forest industry professionals, the results of this review provide baseline information on what has been achieved thus far with UAVs and serve as a guide for using UAVs in forest operations. While UAVs are important tools in modern forest operation activities, they still have challenges and limitations, such as data handling and processing, limited battery life, and the need for standardized protocols that must be addressed. This review provides stakeholders with an integrated understanding of current capabilities and limitations, laying the groundwork for informed decision-making and future innovations in UAV-assisted forest management.

## Data Availability

The datasets used and/or analyzed during the current study are available from the corresponding author on reasonable request.

## Appendix

List of articles reviewed

**Table:**

Author	Title	DOI
(Cheng et al. 2023)	A new method for constructing roads map in forest area using UAV images	10.3233/JCM-226621
(Siafali and Tsioras 2024)	An Innovative Approach to Surface Deformation Estimation in Forest Road and Trail Networks Using Unmanned Aerial Vehicle Real-Time Kinematic-Derived Data for Monitoring and Maintenance	10.3390/f15010212
(Kamarulzaman et al. 2021)	An object-based approach to detect tree stumps in a selective logging area using Unmanned Aerial Vehicle imagery	10.17576/geo-2021-1704-24
(Talbot et al. 2018)	An operational UAV-based approach for stand-level assessment of soil disturbance after forest harvesting	10.1080/02827581.2017.1418421
(Duka et al. 2022)	Application and Accuracy of Unmanned Aerial Survey Imagery after Salvage Logging in Different Terrain Conditions	10.3390/f13122054
(Hruza et al. 2025)	Application of Unmanned Aerial Vehicle and Airborne Light Detection and Ranging Technologies to Identifying Terrain Obstacles and Designing Access Solutions for the Interior Parts of Forest Stands	10.3390/J14f16050729
(Kim et al. 2025)	Assessing Rutting and Soil Compaction Caused by Wood Extraction Using Traditional and Remote Sensing Methods	10.3390/f16010086
(Udali et al. 2022)	Assessing the potential for forest residue classification and distribution over clear felled areas using UAVs and Machine Learning: a preliminary case study in South Africa	10.1109/METROAGRIFOR55389.2022.9964572
(Gejdos et al. 2024)	Assessment of New Techniques for Measuring Volume in Large Wood Chip Piles	10.3390/f15101747
(Sealey and Van Rees 2020)	Assessment of residual slash coverage using UAVs and implications for aspen regeneration	10.1139/juvs-2019-0001
(Briceño et al. 2022)	Assessment of selective logging impacts using UAV, Landsat, and Sentinel data in the Brazilian Amazon rainforest	10.1117/1.JRS.16.014526
(Windrim et al. 2019)	Automated Mapping of Woody Debris over Harvested Forest Plantations Using UAVs, High-Resolution Imagery, and Machine Learning	10.3390/rs11060733
(Türk et al. 2024)	Capabilities of using UAVs and close-range photogrammetry to determine short-term soil losses in forest road cut slopes in semi-arid mountainous areas	10.1007/s10661-024-12339-1
(Türk and Canyurt 2024)	Capabilities of Using UAVs to Determine Forest Road Excavation Volumes in Mountainous Areas	10.31298/sl.148.3-4.3
(Niwa 2025)	Classification of Forest Stratification and Evaluation of Forest Stratification Changes over Two Periods Using UAV-LiDAR	10.3390/rs17101682
(Lepoglavec et al. 2023)	Correct Calculation of the Existing Longitudinal Profile of a Forest/Skid Road Using GNSS and a UAV Device	10.3390/f14040751
(Heppelmann et al. 2022)	Depth-to-water maps as predictors of rut severity in fully mechanized harvesting operations	10.1080/14942119.2022.2044724
(Htun et al. 2024)	Detecting Canopy Gaps in Uneven-Aged Mixed Forests through the Combined Use of Unmanned Aerial Vehicle Imagery and Deep Learning	10.3390/drones8090484
(Udali et al. 2024)	Enhancing precision in quantification and spatial distribution of logging residues in plantation stands	10.1007/s10342-024-01699-5
(Ota et al. 2019)	Estimating selective logging impacts on aboveground biomass in tropical forests using digital aerial photography obtained before and after a logging event from an unmanned aerial vehicle	10.1016/j.foreco.2018.10.058
(Nevalainen et al. 2017)	Estimating the Rut Depth by UAV Photogrammetry	10.3390/J12rs9121279
(Hou et al. 2022)	Estimation of Volume of Felled Chinese Fir Trees Using Unmanned Aerial Vehicle Oblique Photography	10.18494/SAM3996
(Rijal et al. 2023)	Evaluating the feasibility and potential of unmanned aerial vehicles to monitor implementation of forestry best management practices in the coastal plain of the southeastern United States	10.1016/j.foreco.2023.121280
(D’Oliveira et al. 2021)	Impacts of selective logging on Amazon forest canopy structure and biomass with a LiDAR and photogrammetric survey sequence	10.1016/j.foreco.2021.119648
(Sealey and Van Rees 2019)	Influence of skidder traffic on soil bulk density, aspen regeneration, and vegetation indices following winter harvesting in the Duck Mountain Provincial Park, SK	10.1016/j.foreco.2019.01.017
(Kamarulzaman et al. 2022)	Integrated Segmentation Approach with Machine Learning Classifier in Detecting and Mapping Post Selective Logging Impacts Using UAV Imagery	10.3390/f13010048
(Parajuli et al. 2025)	Integrating high-resolution imagery, deep learning, and GIS to estimate soil erosion following timber harvesting	10.1016/j.rsase.2025.101498
(Latterini et al. 2025)	Mapping Skid Trails and Evaluating Soil Disturbance From UAV-Based LiDAR Surveys in Mediterranean Forests	10.1002/ldr.70162
(Bhatnagar et al. 2022)	Mapping wheel-ruts from timber harvesting operations using deep learning techniques in drone imagery	10.1093/forestry/cpac023
(Harvey 2022)	Measuring harvest residue accumulations at New Zealand’s steepland log-making sites	10.33494/nzjfs522022x186x
(Saad et al. 2023)	Modeling Carbon Emissions of Post-Selective Logging in the Production Forests of Ulu Jelai, Pahang, Malaysia	10.3390/rs15041016
(Grube et al. 2025)	Modeling machine-induced soil deformation in forest soils using stump proximity and machine learning	10.1016/j.biosystemseng.2025.104255
(Thiel et al. 2020)	Monitoring Selective Logging in a Pine-Dominated Forest in Central Germany with Repeated Drone Flights Utilizing A Low Cost RTK Quadcopter	10.3390/drones4020011
(Dupuis et al. 2023)	Monitoring selective logging intensities in central Africa with sentinel-1: A canopy disturbance experiment	10.1016/j.rse.2023.113828
(Pszenny 2020)	Possibilities of using UAV and orthophotomosaics to analyze the condition and distribution of operational routes	10.26202/sylwan.2020103
(Hasegawa et al. 2023)	Possibilities of Using UAV for Estimating Earthwork Volumes during Process of Repairing a Small-Scale Forest Road, Case Study from Kyoto Prefecture, Japan	10.3390/f14040677
(Samiappan et al. 2017)	Post-Logging Estimation of Loblolly Pine (*Pinus taeda*) Stump Size, Area and Population Using Imagery from a Small Unmanned Aerial System	10.3390/drones1010004
(Nunes et al. 2021)	Productivity curve models in eucalypt timber forwarding	10.2989/20702620.2021.1936687
(Aquino et al. 2022)	Reliably mapping low-intensity forest disturbance using satellite radar data	10.3389/ffgc.2022.1018762
(Marra et al. 2021)	Remote measuring of the depth of wheel ruts in forest terrain using a drone	10.1080/14942119.2021.1916228
(Dupuis et al. 2025)	Scaling up the assessment of logging’s impact on forest structure in Central Africa using field and UAV data	10.1088/1748-9326/ad99ea
(Alvis et al. 2025)	Spatiotemporal Evolution of Forest Road Rutting and Flow Pathways Examined Using Unoccupied Aerial Vehicles (UAVs)	10.1002/hyp.70105
(Duka et al. 2023)	Terrestrial vs. UAV-Based Remote Measurements in Log Volume Estimation	10.3390/rs15215143
(Kim et al. 2023)	UAV Photogrammetry for Soil Surface Deformation Detection in a Timber Harvesting Area, South Korea	10.3390/f14050980
(Mokros et al. 2016)	Unmanned Aerial Vehicle Use for Wood Chips Pile Volume Estimation	10.5194/isprsarchives-XLI-B1-953-2016
(Goodbody et al. 2017)	Updating residual stem volume estimates using ALS-and UAV-acquired stereo-photogrammetric point clouds	10.1080/01431161.2016.1219425
(Açil et al. 2023)	Use of UAV Data and HEC-RAS Model for Dimensioning of Hydraulic Structures on Forest Roads	10.5552/crojfe.2023.1701
(Papa et al. 2025)	Water and Vegetation as a Source of UAV Forest Road Cross-Section Survey Error	10.3390/f16030507
(López-Amoedo et al. 2023)	Weight estimation models for commercial Pinus radiata wood in small felling stands based on UAV-LiDAR data	10.1016/j.tfp.2023.100436
